# Search for Antiprotozoal Activity in Herbal Medicinal Preparations; New Natural Leads against Neglected Tropical Diseases

**DOI:** 10.3390/molecules200814118

**Published:** 2015-08-04

**Authors:** Núria Llurba Montesino, Marcel Kaiser, Reto Brun, Thomas J. Schmidt

**Affiliations:** 1 Institut für Pharmazeutische Biologie und Phytochemie (IPBP), University of Münster, PharmaCampus, Corrensstraße 48, D-48149 Münster, Germany; E-Mail: n_llur01@uni-muenster.de; 2 Swiss Tropical and Public Health Institute (Swiss TPH), Socinstraße 57, CH-4002 Basel, Switzerland; E-Mails: Marcel.Kaiser@unibas.ch (M.K.); Reto.Brun@unibas.ch (R.B.); 3 University of Basel, Petersplatz 1, CH-4003 Basel, Switzerland

**Keywords:** neglected tropical diseases, antiprotozoal activity, *Plasmodium falciparum*, *Trypanosoma cruzi*, *Trypanosoma brucei rhodesiense*, *Leishmania donovani*, herbal medicinal preparations (HMPs)

## Abstract

Sleeping sickness, Chagas disease, Leishmaniasis, and Malaria are infectious diseases caused by unicellular eukaryotic parasites (“protozoans”). The three first mentioned are classified as Neglected Tropical Diseases (NTDs) by the World Health Organization and together threaten more than one billion lives worldwide. Due to the lack of research interest and the high increase of resistance against the existing treatments, the search for effective and safe new therapies is urgently required. In view of the large tradition of natural products as sources against infectious diseases [1,2], the aim of the present study is to investigate the potential of legally approved and marketed herbal medicinal products (HMPs) as antiprotozoal agents*.* Fifty-eight extracts from 53 HMPs on the German market were tested by a Multiple-Target-Screening (MTS) against parasites of the genera *Leishmania*, *Trypanosoma*, and *Plasmodium*. Sixteen HMPs showed *in vitro* activity against at least one of the pathogens (IC_50_ < 10 µg/mL). Six extracts from preparations of *Salvia*, *Valeriana*, *Hypericum*, *Silybum*, *Arnica*, and *Curcuma* exhibited high activity (IC_50_ < 2.5 µg/mL). They were analytically characterized by UHPLC/ESI-QqTOF-MSMS and the activity-guided fractionation of the extracts with the aim to isolate and identify the active compounds is in progress.

## 1. Introduction

With over a billion people affected worldwide and the health of millions more threatened [3], Leishmaniasis, Human African Trypanosomiasis, and Chagas diseases are considered Neglected Tropical Diseases (NTDs) by the World Health Organization (WHO) because they are neglected by a large part of the pharmaceutical industry and they have low public visibility in high-income countries. NTDs are a group of 17 mostly life-threating or disabling infectious diseases, which are endemic in 149 countries [3], caused by a variety of pathogens such as bacteria, viruses, helminthes, and protozoans.

Although, until relatively recently, the highest number of people affected by NTDs lived in low-income countries in tropical areas of Africa, South America, and Asia, the situation is currently changing and the number of endemic countries with middle-income status is increasing [3]. Furthermore, a potential northward shift of parasite transmission due to climate change from the current range is predicted to concern some European countries and North America in the next decades [4,5]. NTDs constitute, thus, a worldwide public health problem.

The focus of the present project was on the following NTDs: Human African Trypanosomiasis (HAT), and Chagas Disease (caused by species of the genus *Trypanosoma*), and visceral Leishmaniasis (caused by *Leishmania donovani)*. Another tropical protozoan disease, Malaria, caused by species of the genus *Plasmodium*, was also included in the study—even though it is not currently mentioned by the WHO list—due to the high number of infections and deaths that it produces every year.

Considering the very old tradition of plant natural products as remedies against all kinds of disorders, especially infectious diseases, the search for new lead compounds against NTDs in natural sources is a very active field of science [1,2,6,7].

The purpose of the present work was to explore the potential antiprotozoal activity of legally approved and marketed herbal medicinal preparations (HMPs). The use of such HMPs as the source material in the search for new anti-protozoal leads or drugs promises the distinct advantage that they are already in use and thus have been shown to possess relatively few unwanted effects and low toxicity. Furthermore, the starting material is obtained from sustainable biological sources and is easily accessible at relatively low prices, which would represent further advantages in comparison with *de novo* designed chemicals. The positive antiprotozoal activity of such herbal drugs would therefore represent a good starting point for the development of new leads and drugs against protozoal tropical diseases.

## 2. Results and Discussion

### 2.1. Activity Screening of HMPs against Protozoan Parasites

**Figure 1:**
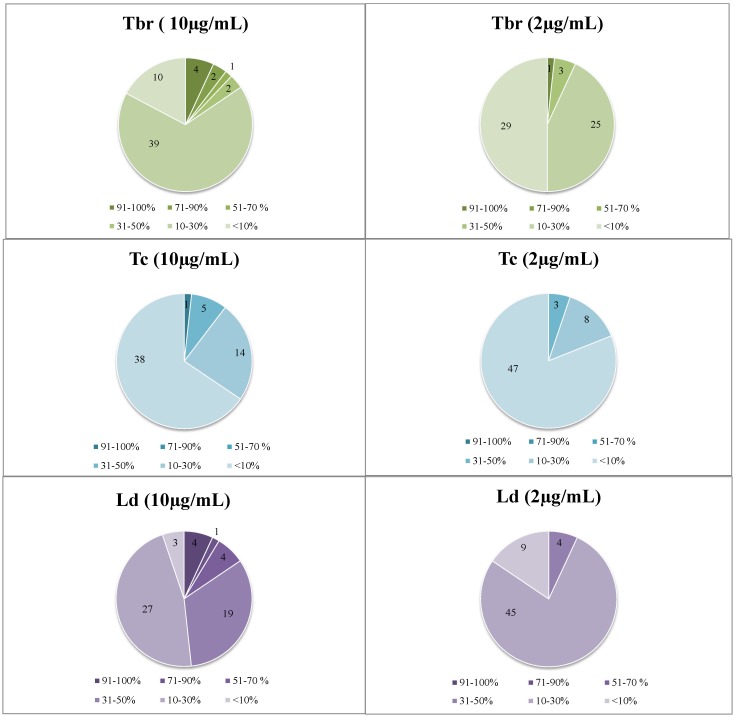

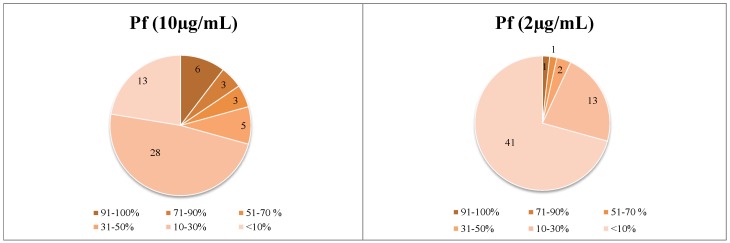
Distribution of the dataset of 58 extracts over the four biological activities under study at the two tested concentrations. Data represent percent growth inhibition of the various parasites at 10 and 2 µg/mL.

A total number of 58 extracts were prepared from 53 HMPs and tested *in vitro* at two test concentrations, 2 and 10 µg/mL, for growth inhibitory (GI) activity against the following parasites: *Trypanosoma brucei rhodesiense* (*Tbr*), *T. cruzi* (*Tc*), *Leishmania donovani* (*Ld*), and *Plasmodium falciparum* (*Pf*), according to the procedures described in Section 3.3. The identity of the HMPs is depicted in Table A1, Appendix. The results of these biological assays are reported in Figure 1 (numerical data are reported in Table A2, Appendix). In case of samples displaying activity in this assay, IC_50_ values against the susceptible parasites as well as for cytotoxicity against mammalian cells (L6 rat skeletal myoblasts) were determined. The results of the IC_50_ determinations and the respective selectivity indices (SI = IC_50_ (L6)/IC_50_ (parasite)) are reported in Table 1. UHPLC-UV chromatograms and LC-MS data of the active extracts, *i.e.*, their suggested dereplicated constituents, are depicted in the section Dereplication Data, Appendix.

**Table 1:** IC_50_ values of active extracts against the most susceptible parasites and for cytotoxicity against L6 cells. All data are expressed in µg/mL and represent the mean of two independent determinations. Highlighted in light green: 2.5 < IC_50_ < 10 µg/mL. Dark green: IC_50_ < 2.5 µg/mL. Orange: SI > 10. Yellow: 10 > SI > 7.5.

ID	*Tbr*	*Tc*	*Ld*	*Pf*	L6	SI (*Tbr*)	SI (*Tc*)	SI (*Ld*)	SI (*Pf*)
***Melarsoprol***	0.004								
***Benznidazole***		0.533							
***Miltefosine***			0.075						
***Chloroquine***				0.002					
***Podophyllotoxin***					0.007				
**2**	n.d.	n.d.	5.34	9.25	67.4			12.6	7.3
**3**	n.d.	n.d.	n.d.	8.21	59.4				7.2
**14**	5.87	n.d.	2.14	0.59	5.28	0.90		2.5	8.9
**22**	n.d.	n.d.	n.d.	10.08	47.0				4.7
**25**	n.d.	n.d.	5.65	2.00	54.6			9.7	27.3
**26**	n.d.	5.87	2.24	4.13	41.6		7.09	18.6	10.1
**40**	n.d.	n.d.	3.17	2.28	47.7			15.1	21.0
**45**	n.d.	n.d.	n.d.	5.83	52.3				9.0
**49**	n.d.	n.d.	4.53	n.d.	50.5			11.1	
20B *	4.03	n.d.	n.d.	n.d.	0.01	0.003			
28B *	3.79	n.d.	n.d.	n.d.	53.7	14.2			
**38**	n.d.	n.d.	n.d.	2.72	55.0				20.3
**39**	1.86	n.d.	n.d.	n.d.	32.3	17.3			
**55**	1.12	n.d.	n.d.	n.d.	12.1	10.9			

* Samples obtained with extraction method B, see Table 2; n.d.: not determined.

Sixteen HMPs showed growth inhibition (GI) activity *in vitro* against at least one of the pathogens (>50% GI at 10 µg/mL), six extracts of which displayed high activity (IC_50_ < 2.5 µg/mL).

Generally, *Plasmodium falciparum* was found to be the most sensitive parasite to the tested HMPs and *T. cruzi* the least sensitive, against which activities hardly ever exceeded 45% of inhibition.

In particular, promising results were obtained with *Arnica montana* and *Salvia officinalis* as the most active preparations against the etiologic agent of East African Human Trypanosomiasis (sleeping sickness). Moreover, the highest antimalarial activities were determined for the extracts of *Curcuma longa*, *Silybum marianum*, and *Hypericum perforatum*. It is noteworthy that the preparation of *Valeriana officinalis* showed antileishmanial activity with an IC_50_ value of 2.1 µg/mL and was the only preparation that also displayed moderate activity against *T. cruzi*. Beside these “first line samples”, some preparations presenting moderate activity (IC_50_ values 2.5–6 µg/mL) and showing high SI values, *i.e.*, ID_38, can still be considered interesting samples which will be subject to following studies.

### 2.2. Antitrypanosomal Activity

The extract of *Arnica montana* L. (Asteraceae) (IC_50_ = 1.12 µg/mL and SI = 10.86) was included in the study as a herbal positive-control, since the anti-trypansosomal activity of its main sesquiterpene lactone constituents was previously described by our group [8,9]. The tincture under study was analyzed by UHPLC/ESI-QqTOF-MSMS and found to contain the main sesquiterpene lactones of the helenalin type known from this plant (see Figure A6 and Table A8, Appendix). Thus, the positive result found with this *Arnica* tincture in the present research is in agreement with the strong antitrypanosomal activity of its major constituents.

With an IC_50_ value of 1.86 µg/mL against *Tbr*, the extract ID_39 of *Salvia officinalis* L. (*Lamiaceae*) appears to be a promising hit for further evaluation. The antitrypanosomal and antimalarial activity of other species of the genus *Salvia* have been previously reported, however, the majority of hitherto isolated and active compounds also showed nonselective toxicity [10,11,12,13,14]. Our tested extract presented a favorable value of SI = 17.3. Therefore, the fractionation of this extract and the evaluation of the resulting biologically active fractions appears interesting. In-depth studies with the aim to identify its antitrypanosomal constituent(s) based on multivariate data analyses in a similar manner, as it was recently described by Ellendorf *et al.* [15], and to target isolation of the relevant natural products are in progress.

The only preparation that showed activity against *T. cruzi* (IC_50_ = 5.86 µg/mL and SI = 7.9) was the ethanolic extract ID_26 of *Valeriana officinalis* L. (Caprifoliaceae—including all former Valerianaceae—[16]). To the best of our knowledge, no previous reports exist on any antitrypanosomal activity of this plant. Even though the biological values of this sample somewhat exceeded our general criteria range for promising activity (IC_50_ < 4 µg/mL and SI > 10), which is probably due to the generally lower sensitivity of this intracellular parasite to drugs, it appears interesting to study extract ID_26 further for the antitrypanosomal activity of its single constituents. Valerian is very widespread in Europe, Asia, and North America and is easy to cultivate, so it might become a promising source of new therapeutics against *T. cruzi* infections.

### 2.3. Antileishmanial Activity

The preparation ID_26 of *Valeriana officinalis* L. also displayed interesting antileishmanial activity. A much higher IC_50_ value (≈225 µg/mL) was previously reported for the chloroform extract of *V. officinalis* [17]. Several isolated constituents of *V. wallichii* were tested against *Leishmania major* and showed activity in a range (from lowest and highest value) but also showed unfavorable toxicity results [18]. In contrast to the published results, our extract displayed an IC_50_ value of 2.1 µg/mL against *Ld* and SI = 18, which constitutes grounds to select this preparation for further evaluation against this parasite.

### 2.4. Antiplasmodial Activity

Extract ID_14, obtained from a commercial preparation of turmeric, *Curcuma longa* L. (*Zingiberaceae*), showed promising antiplasmodial activity with an IC_50_ value of only 0.59 µg/mL, which is significantly lower than previously published data on this plant [19]. In the literature, the rhizomes of different species of *Curcuma* are mentioned to be used in traditional medicine, attributing the therapeutic properties largely to their polyphenolic curcuminoid constituents [19]. Specifically, the most abundant curcuminoid, curcumin, exhibited *in vitro* and *in vivo* antimalarial activity with IC_50_ values ranging from 1.84–3.5 µg/mL [20,21] and an absence of significant toxicity. Furthermore, Arteminisin (ART)-based combination therapies with curcumin have been investigated as a new hope for malaria therapy. Curcumin was found to synergize with ART but also to prime the immune system to protect against recrudescence in mice infected with *Plasmodium berghei* [22]. Based on the significantly lower IC_50_ value of the present extract compared to the published data, experiments are in progress to investigate whether curcumin/curcuminoids are the only active compounds of our extract or whether further synergistic contributions can come from other constituents of the total extract.

The sample ID_25, representing a preparation of Silymarin, a standardized mixture of closely related flavolignans obtained from milk thistle (*Silybum marianum* (L.) Gaertn. (Asteraceae)), showed promising antiplasmodial activity with IC_50_ = 2 µg/mL and SI = 27.3. Milk thistle is one of the oldest medicinal plants and is nowadays used for the treatment of liver damage, hepatitis, and cirrhosis. Moreover, Silybin dihemisuccinate, a derivative of silybin, is used as a clinical antidote for acute *Amanita* mushroom poisoning [23]. In spite of a large number of studies on the pharmacological activity of milk thistle existing in the literature, there is no information, to date, on the antiplasmodial activity of this plant or its constituents, apart from some tests carried out with the synthetized flavolignans 2,3-dehydrosilibinin and 8-(1;1)-DMA-kaempferide [24]. Since the IC_50_ value and SI found in our study were quite favorable and the therapeutic window of the extract is known to be wide, we consider *S. marianum* extract a promising and a useful hit to combat *Pf*. The isolation of the different flavolignans present in the extract and their biological activity tests are in progress.

**Figure A5:**
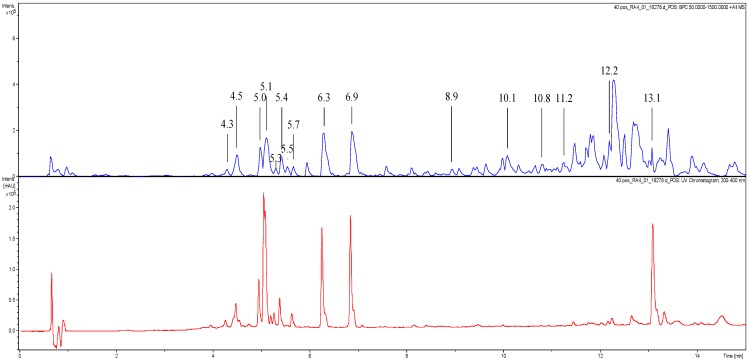
ID_40: Extract of *Hypericum perforatum* L.

**Figure A6:**
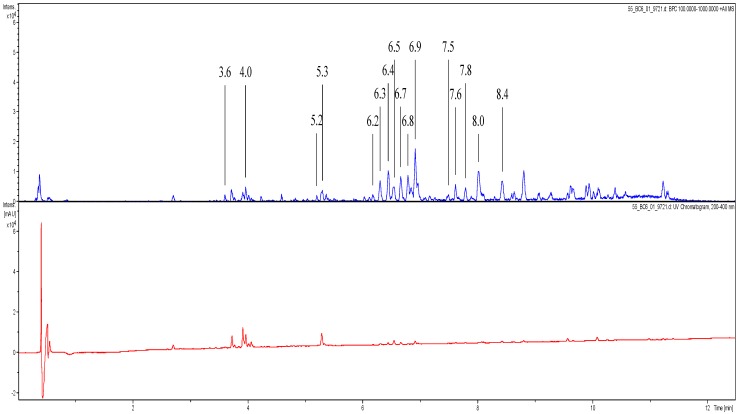
ID_55. Extract of *Arnica montana* L.

The ethanolic extract ID_40 of *Hypericum perforatum* L. (Hypericaceae) showed high activity against *Pf* with an IC_50_ value of 2.27 µg/mL and with favorable selectivity values. Five phloroglucinol derivatives of *H. erectum* were reported to possess antiplasmodial activity [25], however, they were not found in our active extract (see Figure A5 and Table A7, Appendix). Hyperforin, which was present in our sample, and some derivatives were also reported active against *Pf*. For instance, the lithium salt of Hyperforin showed IC_50_ = 2.1 µM [26]. Thus, bioguided fractionation and biological evaluation of isolated compounds compared to the total extract are in progress in order to evaluate whether synergism or the additive effects of single constituents may account for the conspicuous activity of the total extract. Thus, we would justify the use of the complete extract as antiplasmodial instead of the single compounds.

## 3. Experimental Section

### 3.1. Source Material and Reagents

The source material used for this study consists of a selected array of 53 approved or registered HMPs of liquid and solid dosage marketed in Germany and purchased from commercial sources (Table A1, Appendix). Solvents used for extraction procedures and LC-MS determinations were of reagent grade and HPLC grade, respectively.

### 3.2. Extraction Methods

Extraction procedures were applied to (a) lead to an optimal enrichment of potentially active constituents and separate them from unwanted constituents of the galenical matrix and (b), if possible, yield solid extracts that could be directly used for biological testing. Different procedures were applied according to the nature of the preparations (see Table 2 and Table 3).

**Table 2:** Extraction methods used for liquid dosage forms.

ID	Name	Method A			Method B
		EtOAc	Direct Evaporation *	Lyophilization	CH_2_Cl_2_
21	Agnolyt^®^		**X**		
10	Angocin^®^		**X**		
55	Arnikatinktur Hetterich		**X**		
9	Aspecton^®^	**X**			
20	Colchysat^®^		**X**		**X**
42	Eleu Curarina^®^		**X**		
56	Hametum^®^		**X**		**X**
37	Harongan^®^		**X**		
27	Johanniskraut Rotöl ^®^				
32	Koro-nyhadin^®^ Tropfen	X			
35	Legapas^®^		**X**		
34	Misteltropfen Hofmann’s^®^		**X**		
38	Myrrhentinktur Hofmann’s^®^		**X**		
15	Naturreiner Heilpflanzensaft Schwarzrettich			**X**	**X**
44	Nieral^®^ 100		**X**		
8	Prospan^®^	X			
39	Salbei Curarina^®^		**X**		
50	Naturreiner Heilpflanzensaft Brennnessel			**X**	**X**
7	Naturreiner Heilpflanzensaft Huflattich			**X**	**X**
28	Naturreiner Heilpflanzensaft Johanniskraut			**X**	**X**
18	Naturreiner Heilpflanzensaft Löwenzahn			**X**	**X**
4	Tebonin^®^ forte 40 mg	X			
12	Umckaloabo^®^	X			
46	Urophyton^®^ liquidum		**X**		
43	Uvalysat^®^		**X**		

* 96% Ethanol was used as entrainer in case of hydroethanolic and aqueous extracts during evaporation (rotary evaporator).

**Table 3:** Extraction methods used for solid dosage forms.

ID	Name	Extraction Method			
		EtOH	EtOAc	H_2_O	Untreated
31	aar^®^ vir	**X**			
52	Antistax^®^ extra			**X**	
3	Assalix^®^	**X**			
26	Baldrivit^®^ 600 mg	**X**			
47	Bazoton^®^ uno	**X**			
30	Bryophyllum 50%	**X**			
14	Curcu-Truw^®^	**X**			
45	Diufluxx Mono^®^	**X**			
33	Faros^®^ 600 mg	**X**			
24	Femi-loges^®^			**X**	
22	Florafem ^®^	**X**			
16	Gallith				**X**
11	GeloMyrtol^®^/-forte				**X**
41	Ginseng SL				
48	Granu Fink^®^ Prosta forte				**X**
13	Hepar-SL^®^ forte 600 mg			**X**	
29	Hoggar^®^ Balance	**X**			
2	Jucurba^®^ forte 480 mg	**X**			
23	Klimadynon^®^ Uno	**X**			
40	Laif^®^ 900	**X**			
25	Legalon^®^ forte		**X**		
19	Nieron^®^ E 185 mg			**X**	
53	Phlebodril^®^			**X**	
36	Ramend Abführ-Tabletten 20 mg			**X**	
1	Rheuma-Hek^®^ forte 600 mg	**X**			
49	Steiprostat^®^ uno				**X**
5	Styptysat^®^	**X**			
54	Veno SL^®^ 300	**X**			
51	Venostasin^®^	**X**			

#### 3.2.1. Liquid Dosage Form Preparations

Liquid preparations containing low-polarity extracts were obtained by liquid/liquid extraction with low polarity solvents (CH_2_Cl_2_ or Ethyl acetate) and subsequently filtered over Na_2_SO_4_. Extractions were finally concentrated under vacuum using rotary evaporators at 40 °C for large volumes of solvent (>5 mL) or blow-dried with Nitrogen in case of small volumes. Purely aqueous preparations were directly freeze-dried using a lyophilizer.

Some preparations were submitted to two different procedures (methods A and B in Table 2) in order to get a higher range of possible active constituents and facilitate their identification in case of positive biological activity.

#### 3.2.2. Solid Dosage Form Preparations

In the case of hard-shelled capsules and tablets, a number of five pieces or the contents of them were grinded to fine powder and then extracted with 40 mL of the selected solvent (see Table 3) on a magnetic stirrer for 10 min at room temperature. Extractions were vacuum-filtered and concentrated using a rotary evaporator. In case of soft gel capsules containing liquid or oily extracts, their contents was tested without previous extraction.

### 3.3. In Vitro Biological Assays

#### 3.3.1. Multiple-Target-Screening (MTS)

In order to screen the extracted HMPs for antiprotozoal activity, all samples were subjected to a two-concentration (2 µg/mL and 10 µg/mL) multiple target screen, *i.e.*, they were tested for percent growth inhibition (GI) as described previously [9] against the following parasites: *Trypanosoma cruzi* (intracellular amastigotes cultures in L6 rat sekeletal myoblasts as mammalian host cells, Tulahuen C4 strain), *Trypanosoma brucei rhodesiense* (bloodstream trypomastigotes, STIB 900 strain), *Leishmania donovani* (axenic amastigotes, strain MHOM-ET67/L82), and *Plasmodium falciparum* (intraerythrocytic forms, strain NF54).

Samples displaying significantly more than 50% growth inhibition at 10 µg/mL and significant inhibition at 2 µg/mL were considered interesting for further study and, in such cases, the IC_50_ values against the susceptible parasites and cytotoxicity against L6 cells were determined.

#### 3.3.2. IC_50_ Determination and Cytotoxicity Assays

The IC_50_ values of the selected samples yield an exact characterization of their effect against the corresponding parasites. The IC_50_ values were calculated from the sigmoidal growth inhibition curves resulting of a double determination at seven different concentrations [9]. Melarsoprol, benznidazole, miltefosine, and chloroquine were used as a reference drugs for *Tbr*, *Tc*, *Ld*, and *Pf*, respectively.

Cytotoxicity was assessed with a similar IC_50_ protocol using non-infected rat skeletal myoblasts (L6 cells) and Podophyllotoxin as a reference drug [9]. The selectivity index (SI) was calculated as the ratio between the IC_50_ of the L6 cells and IC_50_ of the tested parasites. Those samples that showed at least 10-fold selectivity for the parasites were considered interesting hits for following steps of the study.

### 3.4. Analytical Profiling of the Samples by UHPLC/ESI-QqTOF-MSMS

Analytical characterization of the metabolite profile of the samples was performed by UHPLC coupled with mass spectrometry to obtain detailed information of their constituent profile.

The extracts were injected at a concentration of 2 mg/mL. Chromatographic separations were performed on a Dionex Ultimate 3000 RS Liquid Chromatography System on a Dionex Acclaim RSLC 120, C18 column (2.1 × 100 mm, 2.2 µm) with a binary gradient (A: water with 0.1% formic acid; B: acetonitrile with 0.1% formic acid) at 0.8 mL/min: 0 to 0.2 min: isocratic at 5% B; 0.2 to 9.7 min: linear from 5% B to 100% B; 9.5 to 12.5 min: isocratic at 100% B; 12.5 to 12.6 min: linear from 100% B to 5% B; 12.6 to 15 min: isocratic at 5% B. The injection volume was 10 µL. Eluted compounds were detected using a Dionex Ultimate DAD-3000 RS over a wavelength range of 200–400 nm and a Bruker Daltonics micrOTOF-QII time-of-flight mass spectrometer equipped with an Apollo electrospray ionization source in positive mode at 4 Hz over a mass range of *m*/*z* 100–1000 using the following instrument settings: nebulizer gas nitrogen, 4 bar; dry gas nitrogen, 9 L/min, 220 °C; capillary voltage 4500 V; end plate offset −500 V; transfer time 100 µs; collision gas nitrogen; collision energy and collision RF (Radio Frequency) settings were combined to each single spectrum of 1250 summations as follows: 624 summations with 80 eV collision energy and 130 Vpp + 313 summations with 16 eV collision energy and 130 Vpp + 313 summations with 16 eV collision energy and 130 Vpp. Internal dataset calibration (HPC (High Precision Calibration) mode) was performed for each analysis using the mass spectrum of a 10 mM solution of sodium formiate in 50% isopropanol that was infused during LC re-equilibration using a divert valve equipped with a 20 µL sample loop.

### 3.5. Dereplication of Active Extracts

A partial dereplication of the active extracts was performed by comparison of the obtained UV chromatograms and mass spectra data with the literature and online databases. Moreover, peaks with known *m*/*z* from the literature were also searched using Extract Ion Chromatogram (EIC) monitoring. Nevertheless, some peaks could not be assigned unambiguously and it should be noted that for final assignments, the use of a complementary technique after isolation, such as NMR, would be necessary.

## 4. Conclusions

This screening of a comparatively small number of HMPs led to the discovery of six extracts, namely, from *Curcuma longa* L., *Silybum marianum* (L.) Gaertn, *Valeriana officinalis* L., *Salvia officinalis* L., *Arnica montana* L., and *Hypericum perforatum* L., which showed antiprotozoal activity against at least one of the tested parasites with promising IC_50_ values and a favorable SI, representing a rate of 10% of tested extracts. Seven extracts also showed moderate antiprotozoal activity with IC_50_ values ranging between 2.7 and 5.3 µg/mL. This considerable rate of active hits confirms the interesting potential of HMPs in the search for new antiprotozoal agents. Due to the urgent need of research and development of new therapies against protozoan NTDs and the high efforts necessary to develop new drugs *de novo*, further investigation of the antiprotozoal activity of these and other HMPs may lead to potentially new antiprotozoal compounds of natural origin and facilitate the development of new effective drugs from sustainable and inexpensive natural sources that have already been proven safe.

It should also be considered that natural products in complex mixtures such as plant extracts are often found to show additive or even synergistic effects [27], which could justify the use, in some cases, of the complete extract instead of isolated compounds. Deeper investigations in this direction with the preparations from *Hypericum perforatum* L. and *Curcuma longa* L. are in progress.

## Figures and Tables

**Figure A1:**
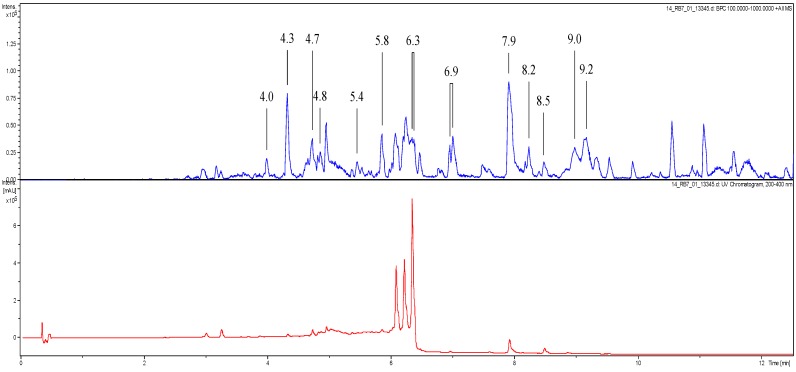
ID_14. Extract of *Curcuma longa* L.

**Figure A2:**
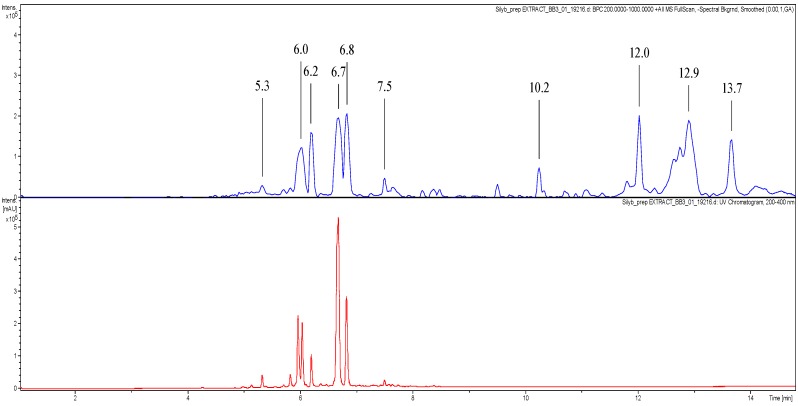
ID_25. Extract of *Silybum marianum* (L.) Gaertn.

**Figure A3:**
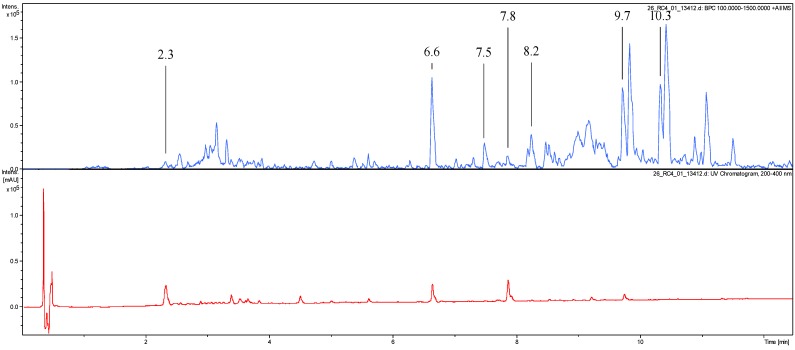
ID_26. Extract of *Valeriana officinalis* L*.*

**Figure A4:**
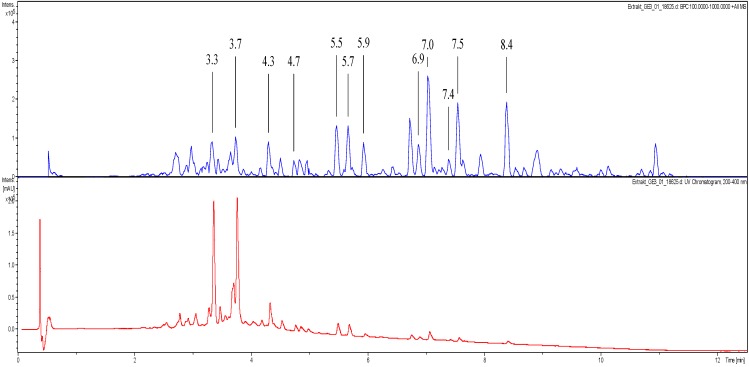
ID_39. Extract of *Salvia officinalis* L.

## Appendix

**Table A1:** Complete dataset of the selected HMPs.

ID	Name	Scientific Name
31	aar^®^ vir	*Echinacea pallida* (Nutt.) Nutt.
21	Agnolyt^®^	*Vitex agnus-castus* L.
10	Angocin^®^	*Marrubium vulgare* L.
52	Antistax^®^ extra	*Vitis vinifera* L.
55	Arnikatinktur Hetterich	*Arnica montana* L.
9	Aspecton^®^	*Thymus vulgaris* L.
3	Assalix^®^	*Salix* sp.
26	Baldrivit^®^ 600 mg	*Valeriana officinalis* L.
47	Bazoton^®^ uno	*Urtica dioica* L.
30	Bryophyllum 50%	*Bryophyllum pinnatum* (Lam.) Oken
20	Colchysat^®^	*Colchicum autumnale* L.
14	Curcu-Truw^®^	*Curcuma longa* L.
45	Diufluxx Mono^®^	*Orthosiphon stamineus* Benth.
42	Eleu Curarina^®^	*Eleutherococcus senticosus* L.
33	Faros^®^ 600 mg	*Crataegus* sp.
24	Femi-loges^®^	*Rheum rhaponticum* L.
22	Florafem ^®^	*Potentilla anserina* L.
16	Gallith	*Glechoma hederacea* L.
11	GeloMyrtol^®^/-forte	Mixture of rectified essential oils of *Eucalyptus globulus* Labill., *Citrus sinensis* (L.) Osbeck., *Myrtus communis* L., *Citrus limon* (L.) Burm.f. (66:32:1:1).
41	Ginseng SL	*Panax ginseng* C.A.Mey.
48	Granu Fink^®^ Prosta forte	*Cucurbita pepo* L.
37	Harongan^®^	*Harungana madagascariensis* Lam. ex Poir.
13	Hepar-SL^®^ forte 600 mg	*Cynara cardunculus* L.
29	Hoggar^®^ Balance	*Passiflora incarnata* L.
27	Johanniskraut Rotöl ^®^	*Hypericum perforatum* L.
2	Jucurba^®^ forte 480 mg	*Harpagophytum procumbens* (Burch.) DC. ex Meisn.
23	Klimadynon^®^ Uno	*Actaea racemosa* L.
32	Koro-nyhadin^®^ Tropfen	*Crataegus* sp*.*
40	Laif^®^ 900	*Hypericum perforatum* L.
25	Legalon^®^ forte	*Silybum marianum* (L.) Gaertner
35	Legapas^®^	*Frangula purshiana* (DC.) J.G.Cooper
34	Misteltropfen Hofmann’s^®^	*Viscum album* L.
38	Myrrhentinktur Hofmann’s^®^	*Commiphora myrrha* (T.Nees) Engl.
50	Naturreiner Heilpflanzensaft Brennnessel	*Urtica dioica* L.
7	Naturreiner Heilpflanzensaft Huflattich	*Tussilago farfara* L.
28	Naturreiner Heilpflanzensaft Johanniskraut	*Hypericum perforatum* L.
18	Naturreiner Heilpflanzensaft Löwenzahn	*Taraxacum officinale* F.H.Wigg.
15	Naturreiner Heilpflanzensaft Schwarzrettich	*Raphanus sativus* L.
44	Nieral^®^ 100	*Solidago virgaurea* L.
19	Nieron^®^ E 185 mg	*Equisetum arvense* L.
53	Phlebodril^®^	*Ruscus aculeatus* L.
8	Prospan^®^	*Hedera helix* L.
36	Ramend Abführ-Tabletten 20 mg	*Senna alexandrina* Mill.
1	Rheuma-Hek^®^ forte 600 mg	*Urtica dioica* L.
39	Salbei Curarina^®^	*Salvia officinalis* L.
49	Steiprostat^®^ uno	*Serenoa repens* (W.Bartram) Small
5	Styptysat^®^	*Capsella bursa-pastoris* (L.) Medik.
4	Tebonin^®^ forte 40 mg	*Ginkgo biloba* L.
12	Umckaloabo^®^	*Pelargonium sidoides* DC
46	Urophyton^®^ liquidum	*Elymus repens* (L.) Gould
43	Uvalysat^®^	*Arctostaphylos uva-ursi* (L.) Spreng.
54	Veno SL^®^ 300	Troxerutin
51	Venostasin^®^	*Aesculus hippocastanum* L.

**Table A2:** Percent Growth Inhibition (GI) values of the tested extracts. Highlighted in Blue: Extracts with 2 < IC_50_ < 10 µg/mL. Green: Extracts with IC_50_ < 2 µg/mL. **A** and **B** indicates those preparations which were prepared with two different extraction methods. (See Table 1 and Table 2).

ID	*Tbr*		*Tc*		*Ld*		*Pf*	
	10 µg/mL	2 µg/mL	10 µg/mL	2 µg/mL	10 µg/mL	2 µg/mL	10 µg/mL	2 µg/mL
**1**	16.8	10.9	46.4	12.3	30.1	8.5	36.3	23.5
**2**	14.6	7.6	16.3	0.0	61.6	23.4	88.3	22.0
**3**	30.1	5.9	0.0	6.4	29.6	22.4	79.1	22.1
**4B**	13.4	4.7	6.8	1.6	21.4	20.6	16.2	0.0
**5**	16.7	13.7	13.5	0.0	31.3	22.4	20.8	16.8
**7A ***	62.1	9.0	0.0	10.5	24.0	17.1	24.7	0.0
**7B ***	21.1	0.0	0.0	2.1	23.4	15.9	21.9	7.4
**8**	8.0	0.0	0.0	6.4	0.8	0.0	20.3	0.0
**9**	16.2	13.7	9.9	1.5	23.9	24.5	9.2	0.0
**10**	15.4	11.2	10.3	12.7	23.4	27.6	13.9	0.0
**11**	9.1	9.4	15.5	0.0	30.2	23.7	18.4	14.7
**12**	15.2	15.7	8.7	0.0	26.0	23.6	8.7	0.0
**13**	23.4	11.4	35.4	30.6	27.6	22.7	23.6	19.2
**14**	80.9	22.3	41.4	0.0	100.0	47.1	99.9	95.4
**15A ***	11.6	49.3	11.3	8.4	22.3	28.0	5.7	0.0
**15B ***	34.4	13.2	7.0	0.0	34.3	15.9	11.9	0.0
**16**	6.7	9.6	0.0	0.0	22.4	22.0	10.0	7.7
**18A ***	11.5	9.2	9.5	8.1	25.6	27.1	16.9	0.0
**18B ***	35.3	12.6	0.0	10.5	32.2	24.1	53.0	0.0
**19**	13.5	5.5	16.9	0.0	17.7	2.4	10.2	5.0
**20A ***	11.3	7.1	47.6	33.8	17.4	25.1	16.8	0.0
**20B ***	99.4	16.3	37.8	37.1	17.5	18.6	33.2	0.0
**21**	4.9	0.7	0.0	5.5	15.6	8.6	8.9	4.4
**22**	28.8	12.1	7.2	0.0	44.8	26.1	73.5	20.9
**23**	15.5	6.2	16.0	7.4	44.8	25.4	30.8	18.2
**24**	6.2	8.0	8.3	4.4	38.5	28.4	5.0	4.4
**25**	28.8	11.0	20.2	8.3	76.0	28.2	99.3	46.5
**26**	17.7	8.8	93.7	31.3	100.0	49.9	99.9	14.5
**27**	10.9	8.8	0.0	0.0	34.1	23.8	8.8	1.2
**28A ***	4.2	5.8	6.8	5.1	0.8	0.0	10.5	0.0
**28B ***	100.0	41.3	13.3	0.0	39.8	24.1	53.0	0.0
**29**	23.6	10.4	0.0	0.0	46.8	20.9	26.2	11.2
**30**	14.3	9.9	0.0	0.0	23.8	6.0	25.3	10.3
**31**	10.8	11.6	0.0	6.6	34.4	26.2	39.9	14.8
**32**	12.7	12.5	0.0	13.3	28.5	22.5	9.4	0.0
**33**	18.3	5.8	0.0	3.8	44.9	26.5	32.8	16.2
**34A**	23.1	11.2	2.9	4.8	36.2	23.6	7.2	0.0
**35**	17.8	12.5	15.5	3.5	31.6	23.3	11.0	0.0
**36**	17.2	12.5	0.0	0.0	34.4	26.5	14.0	10.9
**37**	12.8	8.8	12.4	11.6	27.5	17.6	16.9	0.0
**38**	71.9	14.1	0.0	12.1	67.7	13.4	100.0	56.7
**39**	100.0	39.8	0.0	7.5	48.4	0.0	32.0	0.0
**40**	25.1	19.7	7.3	3.9	93.1	27.7	99.9	35.2
**41**	12.0	12.5	0.0	0.0	26.4	23.4	19.5	6.1
**42**	17.8	9.7	0.0	9.5	24.2	23.0	5.0	0.0
**43**	12.6	10.2	0.4	7.2	26.2	20.6	0.0	0.0
**44**	16.5	9.5	16.6	14.9	54.8	18.3	11.5	0.0
**45**	18.3	10.7	0.4	1.3	40.1	20.3	99.9	18.2
**46**	10.7	13.0	0.0	4.9	24.2	17.8	0.0	0.0
**47**	1.4	8.7	6.2	4.3	22.1	12.2	17.9	0.0
**48**	7.5	11.9	0.0	0.0	6.4	0.0	10.8	8.3
**49**	5.0	7.9	2.0	4.5	63.7	28.9	13.2	2.0
**50**	2.9	0.9	6.9	4.5	18.5	7.0	2.2	0.0
**51**	14.4	8.3	0.0	8.8	40.3	28.2	15.1	5.2
**52**	14.1	4.6	5.7	0.0	41.7	30.7	13.4	0.0
**53**	21.4	4.6	12.4	1.2	38.6	31.0	8.7	0.0
**54**	18.3	8.6	3.1	4.4	44.2	31.9	13.4	0.6
**55**	100.5	92.9	2.6	1.3	91.4	13.7	67.4	0.0

***** HMPs extracted by two different methods, A and B, see Table 2, main document.

## Dereplication Data

Figure A1, Figure A2, Figure A3, Figure A4, Figure A5 and Figure A6. Representative Chromatograms of positive extract preparations dereplicated by UHPLC/+ESI-QqTOF-MSMS. Blue: Base peak chromatogram (*m*/*z* 100–1000). Red: UV Chromatogram 200–400 nm.

Table A3, Table A4, Table A5, Table A6, Table A7 and Table A8. Identification of peaks detected in the extracts.

**Table A3:** Dereplication results for ID_14.

t_R_	Quasimolecular Ion	Suggested Compound
4.0	[M + Na] 275.1628	Unknown
4.3	[M + Na] 275.1634	Unknown
4.7	[M + H] 309.1141 [M + H] 251.1666	Bisdemetoxycurcumin Procurcumadiol or isomer
4.8	[M + Na] 275.1616	Unknown
5.4	[M + H] 251.1635	Procucurmadiol or isomer
5.8	[M + H] 235.1648	Curcumenol/Curcumenone or Isocurcumenol
6.3	[M + H] 369.1335	Curcumin/Cyclocurcumin
6.9	[M + H] 233.1551	Tumeronol A or Tumeronol B
7.9	[M + H] 217.1586	*Ar*-Tumerone/Curzerene or Furanodiene
8.2	[M + H] 279.1587	Unknown
8.5	[M + H] 219.1760	α- or β-Turmerone/Curlone/Germacrone
9.0	[M + H] 333.3003	Unknown
9.2	[M + H] 333.2992	Unknown

**Table A4:** Dereplication results for ID_25.

t_R_	Ion [M + H]^+^	Suggested Compound
5.3	305.0662	Taxifolin
6.0	483.1309	Silychristin/Silydianin *
6.2	499.1253	Unknown
6.7	483.1304	Silybin A/B *
6.8	483.1313	Isosilybin A/B *
7.5	481.1131	2,3-dehydrosilibin
10.2	327.1606	Unknown
12.0	349.2371	Unknown
12.9	n.d.	Unknown
13.7	371.6210	Unknown

* The different flavolignans have the same *m*/*z* and very similar UV-Spectra. The assignments were done in comparison with the literature [28].

**Table A5:** Dereplication results for ID_26.

t_R_	Ion [M + H]^+^	Suggested Compound
2.3	355.1049	Chlorogenic acid
6.6	293.1752	Volvalerenal A or C/Acetoxyvalerenic acid
7.5	520.3419	Unknown
7.8	235.1708	Rulepidol
8.2	279.1574	Unknown
9.7	339.2543	Unknown
10.3	389.3406	Unknown

**Table A6:** Dereplication results for ID_39.

t_R_	Ion [M + H]^+^	Suggested Compound
3.3	463.0897	Luteolin-7-glucuronide
3.7	361.0943	Rosmarinic acid
4.3	287.0573	Luteolin
4.7	271.0610	Apigenin
5.5	315.0864	Cirsimartin
5.5	347.1776	Rosmanol/Epirosmanol isomer
5.7	347.1868	Rosmanol/Epirosmanol isomer
5.9	347.1879	Rosmanol/Epirosmanol isomer
6.9	361.0943	Rosmarinic acid
7.0	331.1932	Carnosol
7.4	375.2183	7α-Acetoxyroyleanone or isomer
7.5	375.2183	7α-Acetoxyroyleanone or isomer
8.4	347.2222	12-methoxy carnosic acid

**Table A7:** Dereplication results for ID_40.

t_R_	Ion [M + H]^+^	Suggested Compound
4.3	579.1541	Unknown
4.5	291.0874	Catechin or Epicatechin
5.0	611.1599	Rutin
5.1	465.1045	Hyperoside/Isoquercitrin
5.3	435.0932	Avicularin
5.4	449.1082	Quercitrin
5.5	373.2232	Unknown
5.7	507.1159	Protohypericin
6.3	303.0518	Quercetin
6.9	539.0992	Biapigenin/I3-II8-biapigenin
8.9	676.3585	Unknown
10.1	587.3946	Unknown
10.8	587.3954	Unknown
11.2	587.3961	Unknown
12.2	496.3299	Unknown
13.1	537.3947	Hyperforin

**Table A8:** Dereplication results for ID_55.

t_R_	[M + H]^+^	Main Fragments	Suggested Compound
3.6	265.1469	247	Dihydrohelenalin
3.6	427.1918	247	Unknown
4.0	479.1140	247	Unknown
5.2	307.1542	247	6-*O*-Acetyl-11α,13-dihydrohelenalin
5.3	305.1376	245	6-*O*-Acetylhelenalin
6.2	333.1742	247	6-*O*-methacryloyl-11α, 13-dihydrohelenalin
6.31	335.1845	247	6-*O*-isobutyryl-11α, 13-dihydrohelenalin
6.4	333.1706	245	6-*O*-isobutyrylhelenalin
6.5	347.1882	247	6-*O*-tigloyl-11α, 13-dihydrohelenalin
6.7	345.1690	245	6-*O*-tigloylhelenalin
6.8	349.2017	247	6-*O*-(2-methylbutyryl)-11α, 13-dihydrohelenalin
6.9	349.2047	247	6-*O*-(2-methylbutyryl)-11α, 13-dihydrohelenalin
7.5	377.1990	291	2β-ethoxy-6-*O*-methacryloyl-2,3-dihydrohelenalin *
7.6	379.2164	291	2β-ethoxy-6-*O*-isobutyryl-2,3-dihydrohelenalin *
7.8	391.2116	291	2β-ethoxy-6-*O*-tigloyl-2,3-dihydrohelenalin *
8.0	393.2331	291	2β-ethoxy-6-*O*-(-2-methylbutyryl)-2,3-dihydrohelenalin *
8.3	335.1852	289	Unknown

* Artefacts due to the reactivity of the helenalin/11,13-dihydrohelenalin derivatives towards the ethanol used in the tincture [29] In the dereplication of this extract, the main fragments resulting from loss of the respective acid moieties are additionally indicated in the table, because they were used for assignment.
